# Mitochondrial D310 mutation as clonal marker for solid tumors

**DOI:** 10.1007/s00428-015-1817-5

**Published:** 2015-08-15

**Authors:** Willemina R. R. Geurts-Giele, Gerard H. G. K. Gathier, Peggy N. Atmodimedjo, Hendrikus J. Dubbink, Winand N. M. Dinjens

**Affiliations:** Department of Pathology, Erasmus MC Cancer Institute, University Medical Center Rotterdam, P.O. Box 2040, 3000 CA Rotterdam, The Netherlands; Service XS, Plesmanlaan 1D, 2333 BZ Leiden, The Netherlands

**Keywords:** Mitochondrial DNA, Tumor clonality, Synchronous tumors, Metachronous tumors

## Abstract

Patients with multiple tumors, either synchronous or metachronous, can have metastatic disease or suffer from multiple independent primary tumors. While proper diagnosis of these patients is important for prognosis and treatment, this can be challenging using only clinical and histological criteria. The aim of the present study was to evaluate the value of mitochondrial D310 mutation analysis in diagnostic questions regarding tumor clonality for a wide range of tumor types. Sanger sequencing of D310 was performed on a diagnostic cohort of 382 patients with 857 tumors that were previously analyzed using routine molecular analysis on genomic DNA. The D310 mononucleotide repeat was frequently somatically mutated (56/321, 17 %) in several tumor types, including breast, head and neck, gynecological, lung, colorectal, and skin tumors. For 84/327 (26 %) patients, a D310 mutation was detected in at least one of their tumors; for these patients, D310 can be used to determine the clonal relationship between their multiple tumors. Clonality assessments based on mitochondrial DNA (mtDNA) and routine genomic DNA analysis were concordant in 52/73 (71 %) patients. We conclude that D310 mutation status might aid in determining clonality of clinically challenging synchronous or metachronous tumors. To this end, next generation sequencing targeted genomic DNA assays should be complemented with mtDNA markers, such as the D310 repeat.

**Electronic supplementary material:**

The online version of this article (doi:10.1007/s00428-015-1817-5) contains supplementary material, which is available to authorized users.

## Introduction

When a patient presents with multiple tumors, either synchronous or metachronous, the question arises whether this is metastatic (recurrent) disease or, alternatively, the patient suffers from multiple primary tumors, as appears to be the case in 8 % of cancer patients [1]. To distinguish between multiple independent primary tumors and metastatic disease is of prime importance for prognosis and treatment [2] but can be challenging, when only clinical and histological criteria are available. Since tumor cells differ from normal cells by the presence of clonal DNA aberrations, these can be used to determine whether or not a clonal relationship exists between multiple tumors within one patient [2–4].

Most molecular clonality assays focus on genomic DNA. Human cells, however, also contain numerous copies of mitochondrial DNA (mtDNA). Mutations in mtDNA initially result in heteroplasmic cells (cells with mutant and non-mutant mitochondrial DNA molecules). Upon cellular expansion, these heteroplasmic cells can achieve mutant DNA homoplasmy (all mtDNA molecules within one cell harbor the same mutation), as has been demonstrated in tumor models, human tumors, and tumor cell lines [5–8]. Apparently, homoplasmic mtDNA aberrations have been frequently found in human tumors [9], notably in a polymorphic cytosine mononucleotide repeat within the non-coding displacement-loop (D-loop) region (D310) [10]. In several studies on different tumor types, mitochondrial DNA alterations have been used as a marker for clonality [11–14]. The aim of the present study was to evaluate for a wide range of tumor types whether or not D310 mutation analysis helps to solve diagnostic questions regarding tumor clonality.

For this study, we selected patients with multiple synchronous or metachronous tumors, for which the question of a clonal relationship was raised leading to routine molecular analysis on genomic DNA. We addressed the following questions: (1) Do these tumors have mtDNA D310 mutations? (2) Are the tumors clonally related based on mtDNA analysis and does this correspond to the clonality status assessed by routine genomic DNA analysis?

## Materials and methods

We studied a cohort of patients with synchronous or metachronous tumors for which routine molecular clonality analysis on genomic DNA had been performed between January 2006 and April 2013 at the Erasmus Medical Center, Rotterdam, The Netherlands. All cases concerned patients for which pathologists or clinicians had previously submitted a request for molecular analysis in view of questions regarding diagnosis, prognosis, and/or patient treatment. For routine analysis, normal and tumor DNA had been extracted from formalin-fixed paraffin-embedded (FFPE) tissue blocks using proteinase K and, for extractions from 2009 onwards, 5 % Chelex 100 resin, as previously described [15]. DNA was used in accordance with the Code of Proper Use established by the Dutch Federation of Medical Scientific Societies (https://www.federa.org/sites/default/files/digital_version_first_part_code_of_conduct_in_uk_2011_12092012.pdf). On these tumors, depending on the amount of tissue available and the tumor type, different combinations of routine molecular analyses had been performed, among which loss of heterozygosity (LOH) analysis, TP53 mutation analysis following abnormal P53 immunohistochemical staining, and/or mutation analysis for other genes.

Of 466 patients eligible for inclusion in the study, 63 were excluded because no archival normal or tumor DNA was available, 17 because the original report was unavailable, and 4 because this was incomplete. In total, 857 tumors from 382 patients were included. Online Resource 1 shows an overview of all tumor details. Consecutive tumors in any single patient included have been numbered T1 to T7, in chronological order with T1 being the first diagnosed; in most cases this was the primary tumor.

PCR amplification of D310 was performed with normal and tumor DNA using Kapa 2G robust hotstart readymix (Kapa Biosystems, Woburn, MA) and M13-tailed custom-made primers (forward TGT AAA ACG ACG GCC AGT - TTG AAT GTC TGC ACA GCC AC and reverse CAG GAA ACA GCT ATG ACC - GGG GTT TGG CAG AGA TGT G). After purification using Exonuclease I and FastAP Thermosensitive Alkaline Phosphatase (Fermentas, Thermo Fisher Scientific, Waltham, MA), PCR products were sequenced with M13 primers using the BigDye Terminator v3.1 kit (Applied Biosystems, Foster City, CA). Fragments were detected on a ABI 3730xl genetic analyzer (Applied Biosystems). D310 repeat length (nucleotide position 303–309) was evaluated by visual inspection using Mutation Surveyor v.3.24 software (SoftGenetics, State College, PA). An altered D310 repeat length in tumor DNA compared to patient-matched normal DNA was classified as a D310 mutation (either deletion or insertion). To exclude genomic DNA amplification, DNA isolated from mtDNA-less cells was used as a negative control (143B/206 ρ0, a kind gift of Dr. G.P. Comi, Dino Ferrari Centre, Neuroscience Section, Department of Pathophysiology and Transplantation (DEPT), University of Milan, Milan, Italy).

## Results

**Fig. 1:**
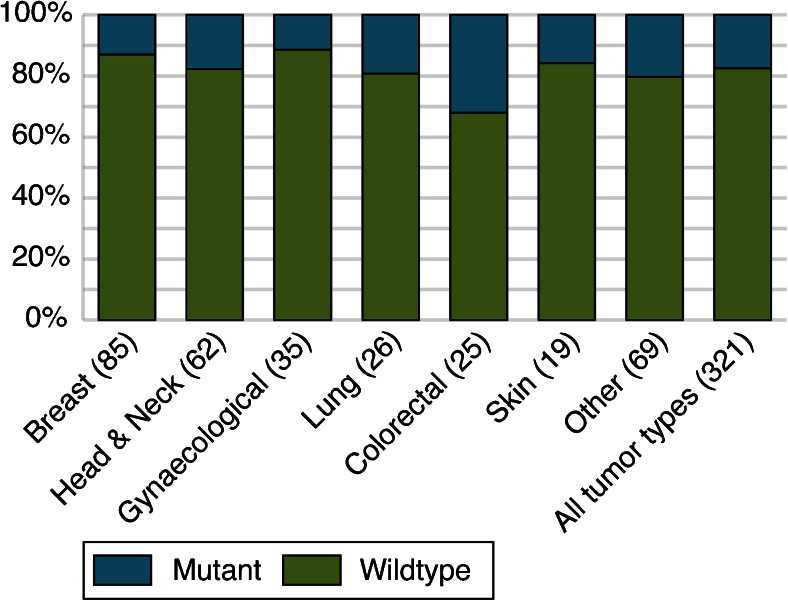
Percentage of D310 mutations in the chronologically first diagnosed tumors of all patients. The tumors are categorized by tumor type; after each tumor type, the number of tumors with an evaluable result is shown *between parentheses*

Detailed results on the analysis of D310 in 857 synchronous or metachronous tumors of 382 patients are shown in Online Resource 1. Corresponding normal DNA could be evaluated in 332 patients and showed D310 repeat lengths of 6, 7, 8, or 9 cytosines (for 1, 187, 123, and 21 patients, respectively). Both normal DNA and DNA from the first tumor (T1) could be evaluated in 321 patients. A D310 mutation was found in 56/321 (17 %) of T1, of which 11/85 (13 %) in breast, 11/62 (18 %) in head and neck, 4/35 (11 %) in gynecological, 5/26 (19 %) in lung, 8/25 (32 %) in colorectal, and 3/19 (16 %) in skin tumors (Fig. 1). In 35/56 (63 %) tumors, an insertion of 1, 2, or 3 nucleotides was found (in 25, 7, and 3 tumors, respectively); 21/56 (37 %) tumors showed a deletion of 1, 2, or multiple nucleotides (18, 2, and 1 tumors, respectively).

**Fig. 2:**
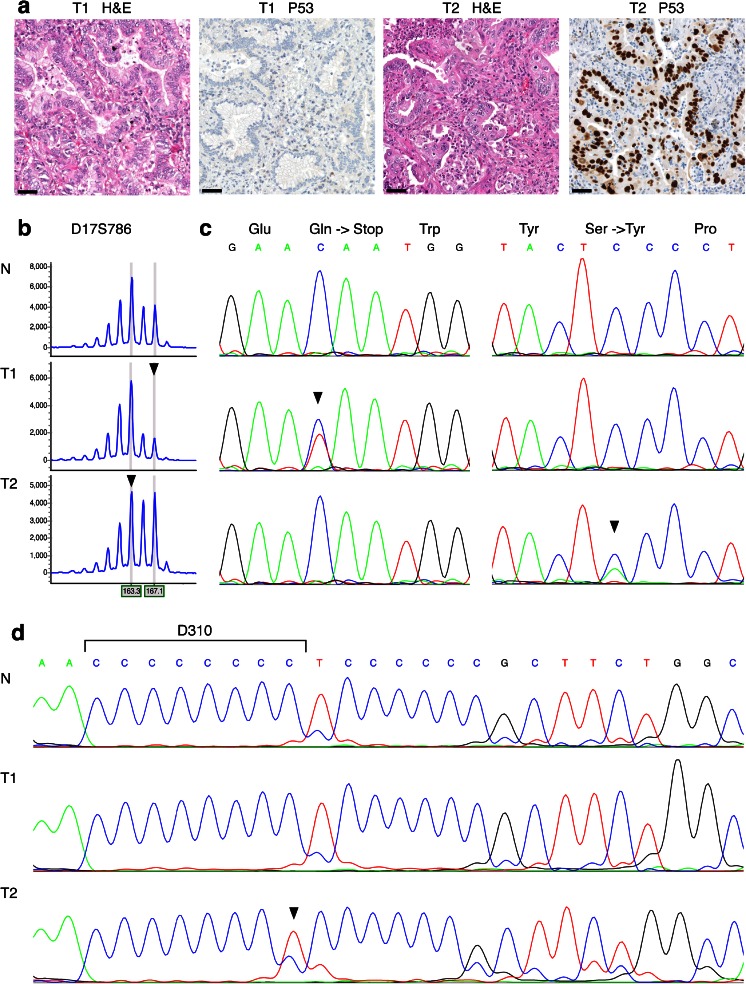
Routine genomic DNA and mitochondrial DNA results for patient 352, whom was diagnosed with synchronous tumors of the right (*T1*) and the left lung (*T2*). **a** Both tumors were diagnosed as adenocarcinomas with a bronchioloalveolar growth pattern; T1 shows absence of P53 staining, whereas T2 shows clear nuclear P53 staining. *Scale bars* represent 50 μm. **b** Routine genomic DNA analysis was performed on DNA isolated from normal (*N*) and both tumor tissues (*T1* and *T2*). LOH analysis of marker D17S786 (TP53) showed loss of the large allele in T1 and loss of the small allele in T2, indicated by *arrowheads*. The horizontal axis indicates the size of the DNA fragments in basepair; the vertical axis indicates signal intensity. **c** Routine Sanger sequencing of TP53 showed a p.Gln52* mutation only in T1, and a p.Ser127Tyr mutation only in T2, both indicated by *arrowheads*. **d** Sanger sequencing of mitochondrial DNA marker D310 showed an 8-cytosine repeat in normal DNA, no aberrations in T1, and a 1-bp deletion in T2, as indicated by the *arrowhead*. The results of routine genomic DNA and mitochondrial DNA analysis both indicate that T1 and T2 represent two primary tumors. *H&E* hematoxylin and eosin stain

**Fig. 3:**
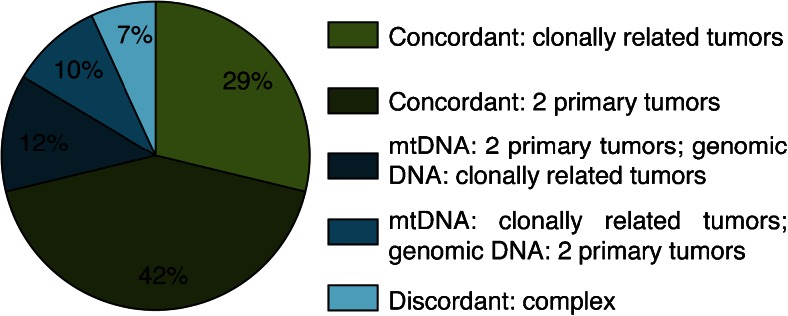
Clonality status assessed by mitochondrial DNA (mtDNA) results compared to routine genomic DNA results for 73 patients with a D310 mutation in one or more of their tumors

Of the 327 patients for whom D310 status could be determined, 243 (74 %) showed tumors without D310 aberrations, while in 84 (26 %) a D310 mutation was detected in one or more tumors (Table 1). Of the 84 patients with a D310 mutated tumor, 29 (35 %) had clonally related tumors and 55 (65 %) had multiple primary tumors based upon D310 mutation status. For 73 of these 84 patients, a final clonality status assessed by genomic DNA molecular clonality analysis was available, and in 52 (71 %), mtDNA and genomic DNA results were concordant (Figs. 2 and 3; Online Resource 2).

**Table 1:** Routine genomic versus mitochondrial DNA results for 84 patients with a D310 mutation in at least one of their tumors

						Genomic DNA results				Mitochondrial DNA results						
Pt	T1	T2	T3	T4	T5	TP53	LOH	Other	Conclusion	N	T1	T2	T3	T4	T5	Conclusion
Concordant: 2 primary tumors																
9	Larynx, 2003	Pleura, 2006				Yes		Yes	T1 ≠ T2	7	7	9				T1 ≠ T2
22	Lung, 1994	Larynx, 2006					Yes		T1 ≠ T2	8	7	8				T1 ≠ T2
25	Breast, 2003	Breast, 2006				Yes	Yes		T1 ≠ T2	8	9	8				T1 ≠ T2
26	Adnex, 2006	Kidney, 2006					Yes		T1 ≠ T2	NE	8	9				T1 ≠ T2
36	Tonsil, 2006	Lung, 2007				Yes	Yes		T1 ≠ T2	8	7	8				T1 ≠ T2
51	Tonsil, 2007	Esophagus, 2007	Lung, 2007			Yes	Yes		(T1 = T2) ≠ T3	8	8	8	9			(T1 = T2) ≠ T3
63	Cervix, 2006	Colon, 2007	Liver, 2007			Yes	Yes		T1 ≠ T3 & T2 ≠ T3	8	8	8	10			(T1 = T2) ≠ T3
64	Lung, 2007	Adrenal gland, 2007					Yes		T1 ≠ T2	8	10	8				T1 ≠ T2
95	Lymph node, 2000	Breast, 2008				Yes	Yes		T1 ≠ T2	8	7	8				T1 ≠ T2
118	Prostate, 2002	Skin, 2008					Yes		T1 ≠ T2	7	7	8				T1 ≠ T2
152	Breast, 1993	Breast, 2009					Yes	Yes	T1 ≠ T2	8	7	8				T1 ≠ T2
155	Breast, 1993	Breast, 2009					Yes	Yes	T1 ≠ T2	9	9	8				T1 ≠ T2
163	Tongue, 2008	Maxilla, 2009					Yes	Yes	T1 ≠ T2	7	7	9				T1 ≠ T2
185	Colon, 2006	Colon, 2010					Yes		T1 ≠ T2	8	7	8				T1 ≠ T2
200	Breast, 2010	Peritoneum, 2010					Yes		T1 ≠ T2	8	8	7				T1 ≠ T2
211	Rectum, 2002	Duodenum, 2010				Yes	Yes		T1 ≠ T2	8	9	10				T1 ≠ T2
213	Larynx, 2005	Esophagus, 2010				Yes	Yes		T1 ≠ T2	8	8	7				T1 ≠ T2
216	Breast, 1998	Bladder, 2010					Yes	Yes	T1 ≠ T2	8	8	9				T1 ≠ T2
232	Breast, 2001	Breast, 2010				Yes	Yes		T1 ≠ T2	8	8	7				T1 ≠ T2
240	Mouth, 2010	Lung, 2010				Yes			T1 ≠ T2	8	9	8				T1 ≠ T2
250	Mouth, 2007	Lung, 2011				Yes	Yes		T1 ≠ T2	9	9	8				T1 ≠ T2
263	Abdomen, 1999	Pelvis, 2011					Yes		T1 ≠ T2	8	7	8				T1 ≠ T2
272	Mouth, 2009	Lung, 2011					Yes	Yes	T1 ≠ T2	8	9	8				T1 ≠ T2
314	Skin, 2011	Skin, 2011					Yes	Yes	T1 ≠ T2	NE	9	8				T1 ≠ T2
315	Vagina, 2011	Liver, 2011	Breast, 2011			Yes	Yes		T1 ≠ (T2 = T3)	8	8	9	9			T1 ≠ (T2 = T3)
352	Lung, 2012	Lung, 2012				Yes	Yes		T1 ≠ T2	8	8	7				T1 ≠ T2
367	Colon, 2012	Lymph node, 2012				Yes			T1 ≠ T2	9	10	7				T1 ≠ T2
368	Lung, 2010	Pancreas, 2012					Yes	Yes	T1 ≠ T2	8	7	8				T1 ≠ T2
371	Breast, 2008	Ovary, 2011	Liver, 2012	Liver, 2012		Yes		Yes	T1 ≠ (T2 = T3 = T4)	8	7	8	8	8		T1 ≠ (T2 = T3 = T4)
373	Breast, 2010	Ovary, 2011				Yes			T1 ≠ T2	8	9	8				T1 ≠ T2
382	Mouth, 2012	Esophagus, 2013				Yes			T1 ≠ T2	7	10	6				T1 ≠ T2
Concordant: clonally related tumors																
6	Nasopharynx, 2006	Maxillary sinus, 2006					Yes		T1 = T2	8	9	9				T1 = T2
41	Breast, 2002	Liver, 2007					Yes		T1 = T2	9	7	7				T1 = T2
59	Skin, 1996	Lung, 2007					Yes	Yes	T1 = T2	8	9	9				T1 = T2
62	Colon, 2004	Colon, 2006					Yes		T1 = T2	9	8	8				T1 = T2
93	Liver, 2006	Liver, 2008					Yes	Yes	T1 = T2	8	7	7				T1 = T2
137	Tonsil, 2008	Nasal cavity, 2009				Yes	Yes		T1 = T2	8	7	7				T1 = T2
143	Lymph node, 2008	Epiglottis, 2008	Lung, 2009			Yes	Yes		T1 = T2 = T3	8	7	7	NE			T1 = T2
177	Esophagus, 2009	Esophagus, 2009				Yes			T1 = T2	7	8	8				T1 = T2
184	Liver, 2007	Colon, 2010					Yes	Yes	T1 = T2	8	10	10				T1 = T2
196	Ovary, 2010	Endometrium, 2010						Yes	T1 = T2	7	10	10				T1 = T2
265	Larynx, 2010	Lung, 2011				Yes			T1 = T2	8	10	10				T1 = T2
270	Lymph node, 2011	Lymph node, 2011					Yes		T1 = T2	8	9	9				T1 = T2
299	Ovary, 2011	Uterus, 2011						Yes	T1 = T2	8	7	7				T1 = T2
326	Colon, 2006	Lung, 2012				Yes			T1 = T2	8	9	9				T1 = T2
344	Scrotum, 2012	Pleura, 2012					Yes		T1 = T2	8	10	10				T1 = T2
347	Skin, 2009	Lung, 2012					Yes		T1 = T2	8	9	9				T1 = T2
362	Breast, 2011	Lung, 2012	Skin, 2012			Yes			T1 = T2 = T3	8	10	10	10			T1 = T2 = T3
365	Breast, 2012	Colon, 2012						Yes	T1 = T2	8	Del	Del				T1 = T2
366	Colon, 2010	Lung, 2012				Yes			T1 = T2	8	9	9				T1 = T2
379	Colon, 2012	Bladder, 2013				Yes		Yes	T1 = T2	8	7	7				T1 = T2
380	Breast, 2011	Skin, 2013					Yes	Yes	T1 = T2	7	8	8				T1 = T2
Mitochondrial DNA: 2 primary tumors; genomic DNA: clonally related tumors																
3	Skin, 2003	Skin, 2003				Yes	Yes		T1 = T2	7	7	8				T1 ≠ T2
12	Breast, 2003	Peritoneum, 2004				Yes	Yes		T1 = T2	NE	8	9				T1 ≠ T2
104	Lung, 2008	Lung, 2008	Liver, 2008				Yes	Yes	T1 = T2 = T3	8	8	9	8			(T1 = T3) ≠ T2
139	Lung, 2007	Lung, 2009					Yes		T1 = T2	8	8	7				T1 ≠ T2
237	Esophagus, 2010	Esophagus, 2010				Yes			T1 = T2	8	9	8				T1 ≠ T2
245	Colon, 2008	Lung, 2010				Yes		Yes	T1 = T2	7	7	8				T1 ≠ T2
268	Larynx, 2010	Lung, 2011				Yes	Yes		T1 = T2	8	8	9				T1 ≠ T2
334	Tonsil, 2008	Lymph node, 2012					Yes		T1 = T2	8	9	8				T1 ≠ T2
350	Larynx, 2010	Larynx, 2010	Lung, 2012				Yes	Yes	T1 = T2 = T3	NE	7	8	7			(T1 = T3) ≠ T2
Mitochondrial DNA: clonally related tumors; genomic DNA: 2 primary tumors																
27	Stomach, 2000	Pancreas, 2006				Yes	Yes		T1 ≠ T2	8	7	7				T1 = T2
219	Lung, 2007	Small intestine, 2010	Small intestine, 2010			Yes			T1 ≠ (T2 = T3)	9	10	10	10			T1 = T2 = T3
248	Breast, 2000	Breast, 2010					Yes		T1 ≠ T2	9	8	8				T1 = T2
262	Breast, 2003	Breast, 2011				Yes			T1 ≠ T2	9	8	8				T1 = T2
275	Pancreas, 2003	Liver, 2011				Yes			T1 ≠ T2	8	9	9				T1 = T2
324	Epiglottis, 2011	Lung, 2012				Yes			T1 ≠ T2	8	9	9				T1 = T2
346	Lymph node, 2011	Palatum, 2012					Yes		T1 ≠ T2	9	10	10				T1 = T2
Discordant: complex																
178	Bladder, 2003	Lung, 2004	Small intestine, 2004				Yes	Yes	T1 ≠ (T2 = T3)	8	8	8	7			(T1 = T2) ≠ T3
187	Oropharynx, 2004	Skin, 2006	Lung, 2010	Lung, 2010		Yes	Yes	Yes	T1 ≠ T2 ≠ T3 ≠ T4	8	8	8	7	7		(T1 = T2) ≠ (T3 = T4)
306	Pharynx, 2011	Mouth, 2011	Larynx, 2011	Lung, 2011			Yes	Yes	T1 ≠ T2 ≠ T4	7	7	8	7	7		(T1 = T3 = T4) ≠ T2
316	Esophagus, 1999	Esophagus, 2002	Esophagus, 2011			Yes		Yes	T2 ≠ T3	NE	7	6	6			T1 ≠ (T2 = T3)
340	Lung, 1990	Bladder, 2007	Breast, 2009	Groin, 2012	Bladder, 2012	Yes			(T2 = T4 = T5) ≠ T3	8	10	8	8	8	9	T1 ≠ (T2 = T3 = T4) ≠ T5
No comparison possible																
46	Breast, 1989	Breast, 2007							No conclusion	8	8	9				T1 ≠ T2
69	Stomach, 2007	Colon, 2007							No conclusion	8	7	8				T1 ≠ T2
91	Tonsil, 2006	Skin, 2008					Yes	Yes	T1 = T2 (uncertain)	8	9	9				T1 = T2
107	Colon, 1995	Colon, 1995	Vertebra, 2008						No conclusion	8	9	9	8			(T1 = T2) ≠ T3
121	Thorax, 1996	Skin, 1998	Breast, 2008						No conclusion	8	8	8	10			(T1 = T2) ≠ T3
126	Breast, 2000	Peritoneum, 2008							No conclusion	8	8	7				T1 ≠ T2
173	Breast, 2009	Breast, 2009							No conclusion	9	7	9				T1 ≠ T2
179	Skin, 2008	Breast, 2009					Yes	Yes	T1 = T2 (uncertain)	8	9	8				T1 ≠ T2
246	Esophagus, 2011	Esophagus, 2011							No conclusion	7	10	7				T1 ≠ T2
259	Ovary, 2011	Endometrium, 2011	Ovary, 2011						No conclusion	8	10	9	10			(T1 = T3) ≠ T2
372	Tongue, 2012	Pleural fluid, 2012						Yes	T1 = T2 (uncertain)	8	9	8				T1 ≠ T2

Patients are categorized according to the final results of both genomic and mitochondrial DNA analyses. Genomic DNA results are based on TP53 mutation analysis, LOH analysis, and/or other analyses (“Yes” indicates that the particular analysis contributed to the final conclusion). Details about the genomic DNA analysis are provided in Online Resource 2. Mitochondrial DNA results show the D310 repeat length for all analyzed tumors

*Del* deletion, *NE* non-evaluable, *pt* patient, *T1-T5* tumor 1-5

## Discussion

We found that the D310 mononucleotide repeat in mtDNA is somatically mutated in 13 % of breast tumors, 18 % of head and neck tumors, 11 % of gynecological tumors, 19 % of lung tumors, 32 % of colorectal tumors, and 16 % of skin tumors. These results are in close agreement with previous studies in which D310 mutations were found in 11–28 % of breast tumors, 0–16 % of head and neck tumors, 3–26 % of ovarian tumors, 0–13 % of lung tumors, and 8–36 % of colon tumors [10].

The identified D310 mutations were (nearly) homoplasmic, indicating that these mutations are present in the majority of the neoplastic cells and as a consequence must have occurred early during oncogenesis. Heteroplasmic D310 mutations have been reported in normal cells, achieving homoplasmy in tumor cells [6, 12]. This suggests that D310 mutation status might provide an ideal marker for tumor clonality. We found in 84/327 (26 %) patients with synchronous or metachronous tumors, for which the question of a clonal relationship was raised, a D310 mutation in at least one of the tumors. In such cases, D310 mutation status can be used to determine the possible clonal relationship between the tumors. In a large majority of patients (71 %), clonality status assessed by mtDNA analysis and routine genomic DNA analysis were concordant.

Discordant results between clonality status assessed by mtDNA and genomic DNA analysis were found in 21/73 (29 %) patients. Clonality assays on multiple tumors often result in some markers with concordant results but also markers with discordant results between the different tumors. Close scrutiny of individual markers is then necessary to decide whether the tumors are clonally related or not in view of the notion that genomic DNA analysis generates a likelihood that multiple tumors might be clonally related, but does not provide a definitive result. For 11 of our patients with discordant results, a highly likely diagnostic result was obtained because the tumors had a mutation in common, had mutually exclusive mutations, or the first tumor had a mutation that was not found in consecutive tumor(s). For these patients, the discordant mtDNA result was probably incorrect. Possible explanations are firstly that two primary tumors by chance may have acquired identical D310 mutations, secondly that de novo D310 mutations acquired during tumor progression result in clonally related tumors with different D310 mutations, and thirdly that intercellular or intracellular heterogeneity (heteroplasmy) in regard of D310 mutations is maintained during tumor development. For five patients, a likely diagnostic result was obtained because a mutation was only present in a consecutive tumor or the tumors showed common or different LOH status of five or more loci. For another five patients, the diagnostic result was weak, based on common or different LOH status of less than five loci. To reliably classify such tumors as clonally related or not, more informative genomic and/or mtDNA markers would be necessary.

Although D310 mutations are the most common mtDNA mutations in human cancer, other mtDNA deletions, insertions, and point mutations have been described [9]. Recently, next generation sequencing assays for mitochondrial DNA have become available [16]. The use of such assays for clonality analysis would result in the detection of more mutations and probably result in a higher predictive value. However, approximately 1.8 point mutations in somatic mtDNA have been found in only 60 % of cancers [9], emphasizing the necessity to include analysis of genomic DNA as well. Mitochondrial DNA markers might be helpful when only a small number of cells are available, in view of the high number of mtDNA copies per cell compared to genomic DNA.

This study also has some limitations. Even though mtDNA is present in numerous copies per cell, facilitating amplification and analysis of a minute number of cells, no or an ambiguous D310 mutation analysis result was obtained for 55/382 (14 %) patients. This was mostly due to an insufficient amount of DNA. For 11/84 (13 %) patients with D310 mutations, a final clonality status assessed by genomic DNA analysis was not available, and for these patients, we were unable to compare mtDNA with genomic DNA results.

We conclude that D310 mutation status might aid in clonality determinations of clinically challenging synchronous or metachronous tumors, but as a single assay, has limited predictive value. To further evaluate the potential contribution of mtDNA markers to assessment of tumor clonality, we propose to include in existing next generation sequencing targeted genomic DNA assays mtDNA markers, such as the D310 repeat.

## Electronic supplementary material

Online Resource 1 (XLS 150 kb)

Online Resource 2 (XLS 59 kb)
